# Fear of heights and mild visual height intolerance independent of alcohol consumption

**DOI:** 10.1002/brb3.162

**Published:** 2013-08-22

**Authors:** Doreen Huppert, Eva Grill, Hans-Peter Kapfhammer, Thomas Brandt

**Affiliations:** 1 Institute for Clinical Neurosciences, Ludwig-Maximilians UniversityMunich, Germany; 2 German Center for Vertigo and Balance Disorders, Ludwig-Maximilians UniversityMunich, Germany; 3 Institute for Medical Information, Processing, Biometrics and Epidemiology (IBE), Ludwig-Maximilians UniversityMunich, Germany; 4 Department of Psychiatry, University Hospital of GrazAuenbruggerplatz 31, 8036, Graz, Austria

**Keywords:** Acrophobia, alcohol consumption, epidemiology, fear of heights, visual height intolerance

## Abstract

**Background** Visual height intolerance occurs when a visual stimulus causes apprehension of losing balance and falling from some height. Affecting one-third of the population, it has a broad spectrum of symptoms, ranging from minor distress to fear of heights, which is defined as a specific phobia. Specific phobias are associated with higher alcohol consumption. This has not been specifically shown for susceptibility to the more general visual height intolerance. **Methods** Representative case–control study nested within a population-based cross-sectional telephone survey to assess epidemiologically 1253 individuals ≥14 years, using a questionnaire on sociodemographic data, typical symptoms, precipitating visual stimuli, and alcohol drinking patterns (overall frequency of alcohol consumption, the daily quantities, and the motives). **Results** Individuals susceptible or nonsusceptible to visual height intolerance showed no significant differences in drinking patterns. The daily average alcohol consumption was slightly higher in persons susceptible to visual height intolerance (4.1 g/day vs. 3.7 g/day). Of those consuming alcohol, cases and controls reported on average consuming 2.3 glasses per day. The prevalence of visual height intolerance was insignificantly higher in the small minority of those drinking 2–3 times per week versus teetotalers. **Conclusions** Our study does not provide evidence that visual height intolerance – contrary to various specific phobias – is significantly associated with individual alcohol consumption patterns.

## Introduction

The association of alcohol drinking patterns and anxiety disorders is well recognized. Evidence indicates that anxiety disorders may cause and aggravate alcohol intake and vice versa (Smail et al. 1984; Himle and Hill 1991; Lotufo-Neto and Gentil 1994; Allan 1995; Kessler et al. 1997; Kushner et al. 2000; Singh et al. 2005; Charriau et al. 2012). The relationship of phobic disorders, especially social anxiety, and alcohol consumption has been emphasized (Morris et al. 2005; Blumenthal et al. 2010; Schneier et al. 2010; Buckner and Matthews 2012). A large representative epidemiological survey in the United States (Stinson et al. 2007) revealed the comorbidity of alcohol abuse and specific phobias. However, patterns of comorbidity vary according to the subtypes of specific phobias (LeBeau et al. 2010; MacDonald et al. 2011); there is a higher comorbidity of animal, situational and blood/injury subtypes than of so-called environmental subtypes (Becker et al. 2007; Depla et al. 2008). Up to 30% of patients with fear of heights sometimes use medication or alcohol for relief (Stransky 1957; Menzies and Clarke 1995; Robinson et al. 2009). The observation by Curlee and Stern (1973) that alcoholics in a psychotherapy group often report fear of heights was not confirmed by others (Ruff et al. 1976) and does not allow generalization.

No systematic study has focussed on the relationship of fear of heights and daily alcohol consumption. Any such association would be relevant for both root cause analysis and the management of these patients. Investigations should take into account that besides the specific phobia fear of heights there is also a more frequent “nonphobic” fear of heights*,* which is best called “visual height intolerance” (Brandt et al. 2012a). Visual height intolerance occurs when a visual stimulus causes apprehension of losing one's balance and falling from some height but without typical symptoms of panic attacks. A recent cross-sectional representative epidemiological study of 3517 individuals reported that the life-time prevalence of visual height intolerance including fear of heights accounts for about one-third of the general population (Huppert et al. 2013). This is the only epidemiological study on “global” visual height intolerance which covered the entire spectrum of symptoms, beginning with a minor distressing height intolerance at one end, then more severe patterns, right up to symptoms of the specific phobia, fear of heights, at the other end. Available epidemiological studies deal with the specific phobia fear of heights (acrophobia) as classified by the ICD-10 (World Health Organization 1993) and DSM-IV TR (American Psychiatric Association 2000) criteria. The life-time prevalence for fear of heights lies between 3.1% and 5.3% (Agras et al. 1969; Curtis et al. 1998; Becker et al. 2007; Stinson et al. 2007; Depla et al. 2008; Oosterink et al. 2009; LeBeau et al. 2010); the life-time prevalence for visual height intolerance is 28% (Brandt et al. 2012b; Huppert et al. 2013).

In the current representative epidemiological study we were interested in how alcohol drinking patterns and the susceptibility to fear of heights and mild visual height intolerance are associated in the general population.

## Material and Methods

### Study design and data collection procedures

A case–control study nested within a population-based cross-sectional telephone survey was conducted. For the survey, a representative sample of individuals aged 14 and above was selected based on a three-stage sampling design. The multistratified, geographically based probability sampling of households allowed an additional random selection of defined targets.

A case was defined as any participant of the survey who reported having life-time visual height intolerance (answering yes to “Have you ever experienced visual height intolerance, an unpleasant feeling caused by visual exposure to heights?”). Controls were selected randomly from the group of participants who did not report ever having had any visual height intolerance. This approach was chosen to minimize selection bias of controls. The study was performed by trained interviewers.

### Measures

The survey questionnaire asked about sociodemographic characteristics, typical symptoms, situations of occurrence, and alcohol consumption.

Presence or absence of visual height intolerance (status as case or control) was defined as outcome. Questions on alcohol consumption were related to the overall frequency of alcohol consumption, the daily quantities of consumed alcohol, and the motive for consuming alcohol, that is, consumption with the intention to relieve visual height intolerance and to cope with the fear-evoking situation. Only cases as defined above answered the latter question. Quantity was defined as glasses of beer, wine or sparkling wine and liquor. Alcohol consumption was calculated on the basis of the reported frequency of consumption and the number of glasses of alcoholic beverages consumed per day. One standard drink was defined as equivalent to 12 g of pure alcohol (World Health Organization 2004). The following categories for alcohol consumption were used: nondrinker, >0–6 g/day (>0–0.5 drink/day), >6–12 g/day (>0.5–1 drink/day), >12–24 g/day (>1–2 drinks/day), >24–60 g/day (>2–5 drinks/day), >60 g/day (>5 drinks/day). As only one person fell in the highest category, the upper two categories were subsumed under one. Heavy alcohol consumption was defined as 60 g/day for men and 30 g/day for women (World Cancer Research Fund/American Institute for Cancer Research 2007).

Sociodemographic characteristics, that is, age, sex, household size, income, occupation, education, and the presence or absence of self-reported fear or panic were used as covariates. Age was stratified into seven categories (<14–19, 20–29, 30–39, 40–49, 50–59, 60–69, 70, and above). Education was stratified into five categories (grade school without vocational training, grade school with vocational training, secondary school, postsecondary school (Abitur) and university, still attending school).

### Statistical analysis

Means were used for continuous variables and percentages for categorical variables. Explorative *t*-tests and chi-square tests were applied for comparisons of individuals with symptoms of visual height intolerance and those without, as well as multiple logistic regression analyses to determine the association of alcohol consumption and visual height intolerance. A variable was a candidate for entrance into the regression model, if it had a *P*-value <0.20 in the bivariate test. To avoid colinearity, variables were selected for entering the multiple logistic models only if the Spearman correlation coefficient was <0.5.

When necessary to choose between two correlated variables, the variable with the stronger association with the dependent variable was entered into the model. As stepwise regression modeling may result in the selection of unstable subsets (Steyerberg et al. 2001), all candidate variables were included in the final models.

SAS statistical software was used for all analyses (V9.3, SAS Institute Inc. Cary, NC). Statistical significance was set at the conventional two-tailed 5% level.

As it had been hypothesized that the association of alcohol consumption and visual height intolerance might be different in persons reporting fear or panic, and that patterns of alcohol consumption might differ in women, the models were also analyzed stratified for self-reported fear/panic and for sex.

## Results

Of a total of 2012 surveyed persons 582 (28.5%) reported a life-time prevalence of height intolerance (visual height intolerance cases, 61.7% women, mean age = 47.6, SD 17.5). Of the remaining 1430 persons without visual height intolerance 683 persons were randomly selected as controls (51.2% women, mean age = 51.2, SD 17.5). Thus, the sample consisted of 1265 persons; 1253 persons answered the questions on alcohol consumption (12 persons refused to answer these questions) (Table 1). Average alcohol consumption was 4.1 g/day for persons with visual height intolerance and 3.7 g/day for persons without visual height intolerance. The difference was not significant. One participant in the visual height intolerance group reported heavy alcohol consumption; no participant in the control group reported heavy alcohol consumption. The daily consumed quantities of alcohol corresponded approximately to data published by the Federal Office of Statistics (Bloomfield et al. 2008). Cases and controls did not differ in alcohol consumption, but in the frequency of alcohol consumption and the daily quantity. The majority in both groups claimed to drink alcohol once a month (30% in cases vs. 31% in controls), followed by two to three times a month (27% vs. 26%); only a small minority reported drinking four times a week or more often (7% vs. 10%). On average, of those consuming alcohol, cases and controls reported consuming 2.3 glasses per day. Three percent of cases reported that drinking alcohol alleviated symptoms of visual height intolerance.

**Table 1:** Sociodemographic characteristics of cases (*n* = 582) and controls (*n* = 683)

Table	*n*	Height intolerance (cases)		No height intolerance (controls)		Total		*P*-value
		
		*n*	%	*n*	%	*n*	%	
Alcohol consumption	1206	582	45.4	683	53.3		100.0	0.2718
nondrinker		114	20.4	141	21.8	255	21.1	
>0–6 g/day (>0–0.5 drink/day)		290	52.0	344	53.1	634	52.6	
>6–12 g/day (>0.5–1 drink/day)		116	20.8	135	20.8	251	20.8	
>12–24 g/day (>1–2 drinks/day)		21	3.8	11	1.7	32	2.7	
>24–60 g/day (>2–5 drinks/day)		16	2.9	17	2.6	33	2.7	
>60 g/day (>5 drinks/day)		1	0.2	0	0.0	1	0.1	
Drinking frequency								0.0696
Never		114	19.6	141	21.0	255	20.4	
Once a month or less		173	29.8	205	30.5	378	30.2	
2–3× per month		157	27.0	174	25.9	331	26.4	
2–3× per week		83	12.4	97	16.7	180	14.4	
4× per week or more		40	6.9	69	10.3	109	8.7	
Fear or panic	1265							<0.0001
Never		340	58.4	526	77.0	866	68.5	
Age in years	1265							0.0004
14–19		45	7.7	55	8.1	100	7.9	
20–29		62	10.7	67	9.8	129	10.2	
30–39		76	13.1	64	9.4	140	11.1	
40–49		127	21.8	125	18.3	252	19.9	
50–59		121	20.8	113	16.5	234	18.5	
60–69		75	12.9	111	16.3	186	14.7	
70+		76	13.1	148	21.7	224	17.7	
Education	1265							0.0008
Grade school without vocational training		16	2.7	38	5.6	54	4.3	
Grade school with vocational training		124	21.3	192	28.1	316	25.0	
Secondary school		146	25.1	125	18.3	271	21.4	
Abitur, university		261	44.8	287	42.0	548	43.3	
Still attending school		35	6.0	41	6.0	76	6.0	
Occupation	1265							0.0125
Employed		350	60.1	363	53.1	713	56.4	
Sex	1265							0.0002
Male		223	38.3	333	48.8	556	44.0	

*N* = 17 answered the question on visual height intolerance with “do not know” and are therefore not included. *N* = 12 refused to answer questions on alcohol consumption.

When covariates were controlled for, neither drinking frequency nor consumed quantity of alcohol were significantly associated with visual height intolerance; however, the prevalence of height intolerance was slightly higher in those drinking 2–3 times per week versus teetotalers. Female sex, age 20–59 versus 70 and over, higher education and self-reported presence of fear or panic were significantly associated with visual height intolerance (Table 2). Stratifying for fear/panic and for sex did not substantially change the results as to the individual alcohol consumption.

**Table 2:** Results of multivariable adjusted model (*n* = 1253) predicting height intolerance (odds ratios >1 indicate higher risk for height intolerance)

Variable	Odds ratio	Lower CL	Upper CL
Female	1.7	1.3	2.2
Age			
14–19 vs. 70+ years	1.5	0.9	2.5
20–29 vs. 70+ years	2.2	1.4	3.6
30–39 vs. 70+ years	2.8	1.7	4.4
40–49 vs. 70+ years	2.2	1.5	3.3
50–59 vs. 70+ years	2.0	1.4	3.1
60–69 vs. 70+ years	1.5	1.0	2.3
Alcohol consumption			
nondrinker vs. >24 g/day (>2 drinks/day)	0.6	0.3	1.2
>0–6 g/day (>0–0.5 drink/day) vs. >24 g/day (>2 drinks/day)	0.6	0.3	1.2
>6–12 g/day (>0.5–1 drink/day) vs. >24 g/day (>2 drinks/day)	0.7	0.3	1.5
>12–24 g/day (>1–2 drinks/day) vs. >24 g/day (>2 drinks/day)	1.5	0.5	4.3
Fear or panic	2.6	2.0	3.3
Higher education (Abitur, university)	1.6	1.2	2.2

CL, 95% confidence limit; d, day; g/day, gram alcohol per day.

## Discussion

The life-time prevalence of visual height intolerance (28.5%) corresponded with findings of our first representative epidemiological study (28%) (Huppert et al. 2013). A significant association between daily alcohol consumption and global visual height intolerance could not be ascertained. There was merely a slightly higher prevalence in those persons who reported moderate, but frequent alcohol consumption (2–3 times a week) compared with those who did not consume any alcohol. The prevalence, however, was not increased in those persons with a greater drinking frequency of four times per week and more. Thus, this result should not be overemphasized. These results correspond to earlier reported lower associations of substance use disorders and environmental specific phobias compared with other specific phobias (Becker et al. 2007; LeBeau et al. 2010). The statistically low significant relationship of subclinical specific phobias and the subsequent onset of alcohol abuse or dependence which MacDonald et al. (2011) described seems contradictory; however, they do not specify the subtypes of specific phobias in their study.

Our failure to find an association of alcohol consumption and visual height intolerance similar to that of alcohol dependence and social anxiety may be due to the fact that height is a peristatic factor that can be easily avoided. Therefore, alcohol is probably not used as a self-medication to cope with expected fearful situations like individuals with anxiety disorders in the general population report (Kushner et al. 2000; Buckner and Matthews 2012) or as especially persons with social anxiety use it to lessen anxiety associated with anticipated specific social situations (Thyer and Curtis 1984; Robinson et al. 2009; Buckner and Matthews 2012). This negative association has consequences for the therapeutic management of persons with visual height intolerance. Therapeutic attention cannot be primarily directed to drinking patterns in contrast to social anxiety disorders (Morris et al. 2005). Treatment strategies should address behavioral aspects of visual height intolerance, for example, habituation by exposition, and improvement of balance control by gaze and locomotion strategies (Brandt et al. 1980). One should also consider that all participants of a qualitative study on visual height intolerance repeatedly reported safety (e.g., anticipatory and actual individual control of visual height stimuli) to be of great importance (Schäffler et al. 2013). These participants referred not only to a general idea of feeling safe but also to the relevance of balance aids such as balustrades, handrails, being on a rope, the quality of hiking shoes, and the importance of having prior experience with a trail.

### Limitation

The major limitation in all studies of this kind is the “self-report of alcohol use”. Although research indicates that this method can deliver a valid index (Kenny and Grant 2007), there can also be some bias, because persons who drink in order to attenuate their symptoms might not be eager to acknowledge this and might underreport their consumption.

## Conclusions

Our study does not provide evidence that visual height intolerance – contrary to various specific phobias – is significantly associated with individual alcohol consumption patterns.
